# Clinical progress and technological innovations in sphincter-preserving treatment for ultra-low rectal cancer

**DOI:** 10.3389/fonc.2026.1685145

**Published:** 2026-02-02

**Authors:** Fan Wu, Xiaojun Shen

**Affiliations:** 1 School of Medicine, Jiangsu University, Zhenjiang, China; 2 Department of General Surgery, Affiliated Kunshan Hospital of Jiangsu University, Kunshan, China

**Keywords:** ultra-low rectal cancer, total mesorectal excision, sphincter-preserving surgery, intersphincteric resection, transanal total mesorectal excision, natural orifice specimen extraction

## Abstract

Ultra-low rectal cancer (defined as a tumor located within 5 cm from the anal verge) poses unique challenges owing to its distinctive anatomical location, necessitating an optimal balance between oncologic safety and functional preservation. This review focuses on the clinical progress and technological innovations in sphincter-preserving management for ultra-low rectal cancer and is organized within a hierarchical framework encompassing oncologic/anatomical principles, surgical procedures, operative approaches/platforms, specimen-extraction strategies, and multimodal therapy. We first outline plane-based resection principles centered on total mesorectal excision (TME) and key aspects of margin quality control. We then systematically summarize the spectrum of sphincter-preserving procedures, including low/ultra-low anterior resection (LAR/uLAR) and reconstructive options such as coloanal anastomosis (CAA), the transabdominal–transanal approach (TATA), pull-through procedures (Bacon and its modifications), and intersphincteric resection (ISR), with comparisons of indications, oncologic safety, and functional outcomes. Furthermore, we discuss the impact of laparoscopic, robotic, and transanal approaches (e.g., TaTME) on deep pelvic exposure, anatomical precision, and the learning curve, as well as the trade-offs between minimally invasive benefits and safety control associated with specimen-extraction strategies such as NOSE. Finally, we summarize the role of neoadjuvant and total neoadjuvant therapy in facilitating sphincter or organ preservation. Overall, sphincter-preserving treatment for ultra-low rectal cancer should be guided by standardized oncologic principles and tailored combinations of procedures and approaches, with the overarching goal of balancing functional benefit against oncologic safety.

## 1 Introduction

Colorectal cancer is the fourth most common cancer and the third leading cause of cancer-related deaths worldwide, with rectal cancer accounting for approximately 35% of cases. The incidence of rectal cancer is notably higher in developed countries (1, 2). Ultra-low rectal cancer is defined as rectal cancer with the tumor located within 5 cm of the anal verge (3). Its low anatomical position makes surgery more difficult and increases the recurrence rate, especially when ensuring radical resection while preserving anal sphincter function. Achieving sphincter-preserving treatment while curing the tumor has become a major challenge in the treatment of rectal cancer. With the improvement in living standards, patients’ expectations for postoperative anal function recovery and quality of life have continuously increased. In recent years, sphincter-preserving treatments have made significant technological and conceptual advancements, greatly reducing postoperative complications and improving patients’ quality of life. This article provides a brief overview of the clinical progress in sphincter-preserving treatment for ultra-low rectal cancer.

## 2 Oncologic and anatomical principles

Ultra-low rectal cancer is typically located in the distal rectum, near the anorectal junction, often at or below the level of the peritoneal reflection and is closely associated with the sphincter complex. At this level, the operative workspace is markedly constrained, and anatomical dissection must strictly respect several key structures, including the anorectal ring, the levator ani muscle, and the internal and external anal sphincters. In 1982, Heald (4) and colleagues first introduced the concept of Total Mesorectal Excision (TME), marking a milestone innovation in rectal cancer surgery. TME follows the principle of precise fascial anatomy and comprehensive excision, advocating for the total removal of the rectum along with its surrounding mesorectal fat, lymph nodes, and blood vessels in rectal cancer surgery. This approach ensures the prevention of tumor spread and reduces the risk of local recurrence, while excision should be performed along the anatomical plane of the mesorectum (also known as the holy plane). Between the outer membrane of the rectum and its mesorectum and the surrounding pelvic wall, there exists a natural, avascular anatomical space called the holy plane. In this plane, the mesorectum can be removed as completely as possible while avoiding damage to nerves and blood vessels (4–6). As its application has deepened, the TME principle has gradually become the “gold standard” for rectal cancer surgery (7). By adhering to fascial anatomy, TME not only lowers the local recurrence rate but also reduces wound bleeding and exudation, and the clearer surgical field enhances the sphincter-preserving rate in ultra-low rectal cancer surgeries.

## 3 Surgical procedures

### 3.1 Abdominoperineal resection

Abdominoperineal resection (APR) originated in the early 20th century and was proposed by the renowned surgical pioneer Miles (8) in 1908, known as Miles APR. This procedure provided a new approach to radical resection for low rectal cancer and laid the foundation for subsequent rectal cancer surgeries. Miles APR advocates for maximal radical resection, removing the rectum, anal canal, and surrounding tissues during surgery to ensure tumor radicality. Although it can reduce the risk of local recurrence, the procedure involves significant trauma and requires the creation of a permanent colostomy, leading to a high incidence of postoperative complications and severely affecting the patient’s quality of life (8–10). Traditional radical surgery for ultra-low rectal cancer typically uses a blunt separation method in the presacral space, which not only makes it difficult to ensure the integrity of the rectal fascia but also complicates the separation of the puborectal muscle space due to bleeding and poor exposure. As a result, cases where the tumor is located less than 7 cm from the anal verge often struggle with sphincter preservation (8). Pathological specimens from traditional APR surgeries often reveal many tumors with a lower margin far from the anal verge, or tumors infiltrating T2 or below, which is a missed opportunity for sphincter preservation. With the global adoption of the total mesorectal excision (TME) principle, most medical institutions now perform modern abdominoperineal resection (APR) following the precise TME plane. A prospective controlled study compared the safety and oncological outcomes of ELAPR, which has clear anatomical landmarks, with traditional APR in treating low rectal cancer. The results showed that Blood loss (P = 0.021), perineal wound complication (P = 0.039), intraoperative perforation (IOP) rate (P = 0.028), local recurrence (P = 0.034) were significantly less frequent in the ELAPR group. There was greater circumferential resection margin (CRM) involvement in the conventional APE group but no statistical difference between the two groups (11). This indicates that the advancement of the TME principle has significantly improved the quality of traditional APR, reducing the circumferential margin positivity rate and the risk of local recurrence.

### 3.2 Ultra-low anterior resection with coloanal anastomosis

#### 3.2.1 The Dixon procedure

The Dixon procedure (also referred to as low anterior resection, LAR) was first reported by Claude F. Dixon in 1948. Its central concept is to restore bowel continuity while achieving oncologically radical resection for rectal cancer, thereby avoiding a permanent stoma. In contemporary colorectal surgical practice, “Dixon” generally corresponds to the anterior resection spectrum (LAR/uLAR), namely, performing rectal resection with subsequent colorectal or coloanal reconstruction to preserve anal sphincter function, provided that adequate resection margins and oncologic radicality can be ensured (12). With the widespread adoption of the TME principle, plane-based resection and specimen quality control have become increasingly standardized, further strengthening oncologic safety for this group of procedures. To address the technical constraints of deep pelvic work during the distal TME phase of uLAR, the “Gate approach” has been proposed to facilitate safer distal anterolateral pelvic dissection within the TME plane and to support pelvic autonomic nerve preservation (13). Nevertheless, low anastomosis–related complications remain a major limiting factor, particularly anastomotic leakage. In a classic cohort of patients undergoing LAR plus TME, the rate of severe anastomotic leakage was approximately 11.0%, with severe leaks occurring predominantly in patients with an anastomotic height <6 cm (14). In addition, functional outcomes after Dixon warrant close attention. An international consensus defines low anterior resection syndrome (LARS) as bowel dysfunction following rectal resection that adversely affects quality of life and recommends standardized instruments for clinical assessment and follow-up (15). In recent years, reconstructive strategies have continued to evolve, for example, transanal transection with single-stapling reconstruction (TTSS) has been proposed, with early results suggesting potential advantages in reducing perioperative morbidity while maintaining oncologic integrity (16).

#### 3.2.2 The Parks procedure

Coloanal anastomosis (CAA) is an important reconstructive option after sphincter-preserving resection for low and ultra-low rectal cancer. The classic technique can be traced back to the concept of transanal low anastomosis proposed by Parks in 1972, which enabled restoration of bowel continuity and avoidance of a permanent stoma even after ultra-low rectal resection (17). In the setting of uLAR, when the distal rectal stump is too short to allow a conventional anastomosis, CAA—particularly a hand-sewn anastomosis—may serve as a key reconstructive choice. It remains associated with an increased risk of postoperative bowel dysfunction, including higher stool frequency, urgency, and fragmented defecation. To mitigate defecatory dysfunction resulting from loss of rectal reservoir capacity, Parc and colleagues introduced colonic J-pouch reconstruction on the basis of Parks CAA, and subsequent studies have shown that a J-pouch can improve bowel function and quality-of-life–related measures during certain follow-up periods (18–20). In recent years, further comparative studies have evaluated reconstructive strategies for ultra-low anastomoses, including comparisons of hand-sewn CAA with other ultra-low anastomotic techniques in terms of functional outcomes, underscoring the need for individualized reconstructive decision-making based on tumor location, sphincter status, and patient expectations (21, 22).

#### 3.2.3 Transanal abdominal transanal

In the context of ultra-low anterior resection requiring coloanal anastomosis, achieving sufficiently low and safe distal transection and precise anastomosis within the confines of a narrow, deep pelvis represents one of the key technical challenges in sphincter-preserving surgery. The Transanal Abdominal Transanal (TATA) is a representative workflow that was developed in response to this need. The TATA procedure, proposed by Dr. Gerald Marks in 1984, marked a turning point in the history of sphincter-preserving surgery for rectal cancer. In the treatment of rectal cancer, traditional APR can effectively remove tumors but often necessitates the creation of a colostomy, resulting in the loss of normal bowel function (8–10). As a result, the TATA procedure was developed, utilizing a dual approach through both the abdomen and anus, ensuring safe and complete tumor resection while preserving anal function as much as possible, thus avoiding the need for a permanent colostomy (23). TATA initially faced limitations due to its technical complexity, it laid the foundation for future innovations in sphincter-preserving surgery, particularly influencing the development of more advanced techniques such as Transanal Total Mesorectal Excision (TaTME) and Transanal Endoscopic Intersphincteric Resection (TaE-ISR).

### 3.3 Pull-through procedure (Bacon procedures and their modifications)

The Bacon procedure is one of the classic sphincter-preserving operations for rectal cancer. Its central concept is that, after resection of the rectal tumor, the proximal colon is everted and pulled through the anus and managed in a staged manner to restore bowel continuity and avoid a permanent stoma. Bacon and colleagues systematically described the feasibility and oncologic outcomes of this procedure in selected patients with rectal cancer (24). Subsequent studies have suggested that the indications for the traditional Bacon pull-through are relatively limited; however, with careful patient selection, its overall efficacy may be comparable to that of low anterior resection or APR (25). To address even more “ultra-low” lesions and clinical scenarios in which the distal stump is extremely short and an intra-abdominal anastomosis is difficult to perform, a variety of modified Bacon techniques (e.g., optimization of the staging strategy and colonic management) have been proposed with the aims of shortening hospitalization, improving anorectal function, and expanding the applicability of sphincter preservation (26, 27). In recent years, the two-stage Turnbull–Cutait delayed coloanal anastomosis (DCAA) has attracted renewed attention. Its theoretical advantages include reducing the risk of anastomotic leakage and, in selected patients, decreasing the need for or even avoiding a diverting stoma. Available studies indicate that two-stage pull-through reconstruction offers acceptable perioperative safety and oncologic feasibility for reconstruction after low/ultra-low rectal cancer resection, although individualized selection remains necessary, taking into account the costs and logistics of staged management as well as postoperative bowel function (28–30).

### 3.4 Intersphincteric resection

**Figure 1:**
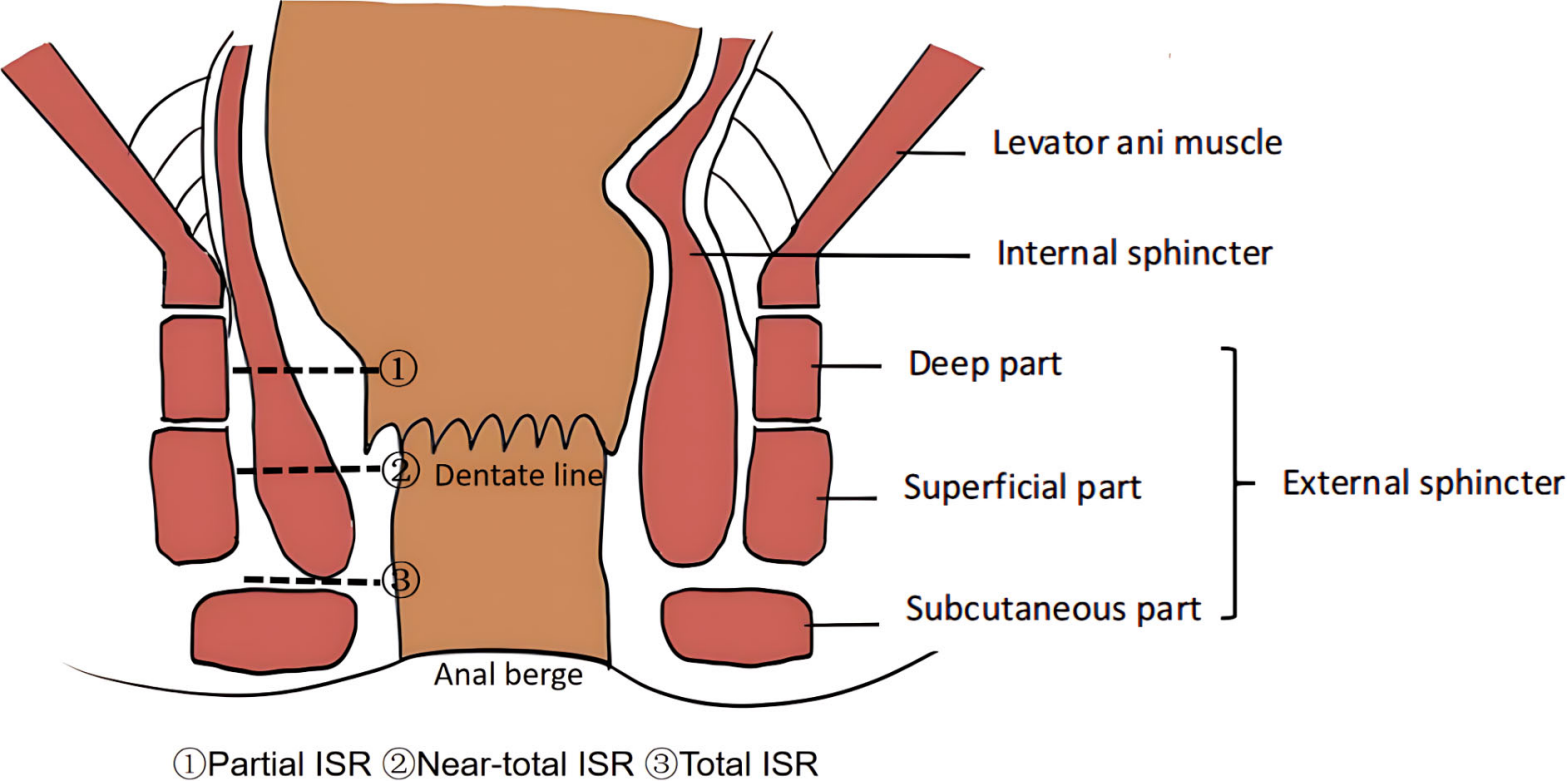
Schematic diagram of intersphincteric resection (ISR) for ultra-low rectal cancer. The image illustrates the different levels of resection in ISR, including Partial ISR (1), Near-total ISR (2), and Total ISR (3). Key anatomical structures such as the anal verge, dentate line, internal and external sphincters, and levator ani muscle are labeled. The internal sphincter is further divided into the deep, superficial, and subcutaneous parts, while the external sphincter is highlighted on the right. This diagram helps visualize the extent of sphincter involvement during the resection procedures in ultra-low rectal cancer surgeries.

Intersphincteric resection (ISR) was first proposed by Schiessel et al. (6) in 1994, aiming to provide sphincter-preserving treatment for patients with ultra-low rectal cancer who would traditionally require the sacrifice of anal function (as in abdominoperineal resection). This technique marks the shift in rectal cancer surgery from “radical cure” to “functional preservation.” Currently, based on the extent of internal anal sphincter resection, ISR is generally categorized into three types (**Figure 1**): (1) Partial ISR: only the upper third of the internal anal sphincter is removed, suitable for tumors located higher in the rectum that have not widely invaded the internal anal sphincter; (2) Near-total ISR: approximately two-thirds of the internal anal sphincter is removed, suitable for tumors located lower but confined to part of the internal anal sphincter; (3) Total ISR: the entire internal anal sphincter is removed, suitable for tumors that almost involve the entire internal anal sphincter (31, 32). ISR surgery primarily utilizes the anatomical space between the internal and external anal sphincters for resection. The surgery requires precise separation of anatomical planes, removing the affected internal anal sphincter while preserving the external anal sphincter and pelvic nerves, thereby retaining partial sphincter function and avoiding the need for a permanent stoma. This approach significantly improves patients’ postoperative quality of life. Studies show that approximately 73% of patients undergoing ISR experience Low Anterior Resection Syndrome (LARS), compared to about 58% in standard low anterior resection surgeries and around 38% in partial rectal resections (33). This indicates that, as the internal anal sphincter is crucial for maintaining resting pressure and controlling defecation, partial or total resection can lead to increased post-operative defecation frequency, urgency, and some degree of fecal incontinence. A study by Japanese researchers (34) involving 2,125 patients who underwent ISR showed relatively low mortality and morbidity, with good survival rates; local recurrence rates and postoperative fecal incontinence were somewhat higher. Compared to APR (35), both procedures show similar local control and complication rates, ISR avoids the permanent stoma, significantly improving patients’ quality of life, despite the possibility of post-operative defecation dysfunction.

In the treatment of ultra-low rectal cancer, laparoscopic ISR, transanal ISR and robot-assisted ISR are all clinically valuable sphincter-preserving surgical options. These surgical approaches each have unique advantages in the treatment of ultra-low rectal cancer (36). Further details are provided in Chapter 4. A comprehensive evaluation based on the patient’s specific condition and tumor location is necessary, and the future application of these techniques holds great promise.

In recent years, the combination of TaTME with ISR, known as Transanal Endoscopic Intersphincteric Resection (taE-ISR), has attracted considerable attention. The transanal endoscopic approach provides a good field of view for performing intersphincteric resection, overcoming the poor visibility issues of traditional ISR (37). Xu Xi et al. demonstrated that compared to the ISR group, the TaE-ISR group had lower rates of adjacent organ injury (0.0% vs. 5.6%, P = 0.059), distal margin positivity (1.1% vs. 8.9%, P = 0.034), and incomplete specimens (2.2% vs. 13.3%, P = 0.012). The anal preservation rate in the TaE-ISR group was significantly higher (97.8% vs. 82.2%, P = 0.001). Patients in the TaE-ISR group had lower disease-free survival (P = 0.044) and cumulative recurrence rates (P = 0.022) compared to the ISR group. This technique significantly improves surgical quality, anal preservation, and survival outcomes for patients with ultra-low rectal cancer (37, 38). This result indicates that integrating TaTME technology into ISR has potential clinical value and may further improve the sphincter preservation success rate for ultra-low tumors.

The development of ISR surgery has evolved from theory to practice, from a single technique to a standardized and personalized treatment approach (39). This progress has not only been driven by advancements in surgical techniques but also by developments in imaging, science, and technology, as well as improvements in postoperative management. ISR surgery may still lead to postoperative complications such as bowel control disorders and fecal incontinence, compared to traditional treatments like APR, it has significantly improved patients’ quality of life. Today, ISR surgery has gradually become an essential surgical method for treating ultra-low rectal cancer (40).

## 4 Operative approaches and platforms

### 4.1 Laparoscopic approach

With advancements in laparoscopic equipment and techniques, the high-definition magnified vision, minimally invasive nature, and thorough lymphadenectomy have led to the global recognition and widespread adoption of laparoscopic gastrointestinal cancer surgeries. In rectal cancer surgery, the unique visualization and meticulous precision of laparoscopy, combined with the principles of total mesorectal excision (TME) for rectal cancer, have significantly improved the anal preservation rate in ultra-low rectal cancer. Moreover, its oncological outcomes are comparable to those of open surgery (41, 42). With the advancement of minimally invasive techniques, laparoscopic ISR has gradually become a popular option. Laparoscopic ISR is performed through an abdominal incision, supplemented by laparoscopic technology, aiming to preserve anal function while completely excising the tumor. This approach is commonly applied to patients with ultra-low rectal cancer where the tumor is located less than 5 cm from the anal verge or less than 3 cm from the dentate line, typically for those diagnosed with T1, T2, and N0–1 stages (43). Park et al. (3) conducted a comparative study on the outcomes of open surgery and laparoscopic surgery in 210 cancer patients. The results showed similar rates of major complications between the two groups (5.4% vs. 3.8%; P = 0.428). During the 34-month follow-up period, patients in the laparoscopic group experienced significantly shorter hospitalization and recovery times for defecation, a reduction in operative time by 16 minutes (P = 0.230), and less intraoperative blood loss (P = 0.002). These findings indicate that compared to traditional open surgery, laparoscopic ISR offers advantages such as smaller incisions and faster recovery. laparoscopic ISR is associated with less bleeding, lower postoperative recurrence rates, and better anal preservation outcomes (44).

### 4.2 Robotic approach

With the continuous development and popularization of robotic technology, robotic-assisted ISR (Robotic-assisted Intersphincteric Resection, RISR), such as the Da Vinci surgical robot, has gradually entered clinical practice (45). For cases with narrow pelvises, obesity, or complex surgical difficulties, robotic assistance offers higher flexibility and precision (46, 47). In ultra-low rectal cancer surgeries, the main advantages of robotic surgery include flexible robotic arms that facilitate operations in narrow pelvic spaces, stable exposure provided by the mechanical arms, and 3D high-definition magnified vision that aids in distinguishing tissue structures. Experience from several centers shows that robotic-assisted ISR significantly increases the probability of successful sphincter preservation compared to open surgery (OR ≈ 3.47), allowing more patients to undergo total ISR instead of being forced to switch to a permanent stoma (48). Despite its technical advantages, the clinical outcomes of robotic surgery have not clearly outperformed traditional laparoscopy (46). Research by Yoo BE et al. (49) showed similar results in terms of postoperative complications, positive margin rates, and survival between robotic-assisted and laparoscopic surgery, with no statistically significant differences. Robotic surgery is currently associated with high costs and a long learning curve, which may limit its widespread adoption in routine practice. For patients with difficult pelvic anatomies or obesity, robotic assistance offers significant advantages over traditional laparoscopic surgery, particularly in terms of precision and maneuverability. Despite these benefits, the short-term advantages remain debated, and further stratified studies are needed to fully assess its impact on patient outcomes. With advancements in surgical robot development and reductions in robotic arm costs, robotic-assisted surgery is expected to be the future direction of minimally invasive surgery, benefiting a wider and larger patient population in the future.

### 4.3 Transanal approach

Transanal total mesorectal excision (TaTME) is a minimally invasive technique performed via a transanal approach and has developed rapidly in recent years in the field of rectal cancer treatment (50). Because low rectal cancer surgery requires extremely precise surgical fields, traditional abdominal TME is often limited by factors such as a narrow pelvis and specific patient body types (e.g., male or obese patients) (9, 51, 52). These limitations make it difficult for surgeons to achieve ideal visibility during resection, thereby affecting the surgical margins and local recurrence rates. TaTME overcomes these challenges, with its core advantage being the direct access to the mesorectal space via the transanal approach, utilizing the transanal platform to establish pneumoperitoneum. This significantly improves the surgical field of view, allowing for precise excision of the tumor area under endoscopic guidance, ensuring sufficient safety margins and increasing the success rate of radical tumor resection (53). During the procedure, TaTME relies on high-level endoscopic skills and team collaboration. The surgery can begin via the transanal approach or with both transanal and transabdominal laparoscopic routes simultaneously: after pneumoperitoneum is established, a circular incision is made on the rectal wall approximately 2 cm from the tumor, and the rectal mesorectal space is carefully dissected upwards towards the abdominal cavity following the total mesorectal excision principles. Meanwhile, the transabdominal laparoscopic approach frees the sigmoid colon, upper rectum, and corresponding mesentery, continuing downwards to meet the transanal route, ultimately completing the bowel division and gastrointestinal reconstruction (54). Despite its oncological advantages (53), TaTME has certain drawbacks. The surgery follows an upward approach, which is quite different from traditional surgeries, and the unfamiliar anatomical view requires high technical expertise from the surgeon. The learning curve is steep, and it demands a deeper understanding and proficiency in transanal endoscopic operations and pelvic anatomy by the surgical team. If not mastered properly, there is a risk of injury to the urethra and neurovascular bundles, increasing postoperative complications (55). Several studies suggest that improper technique or insufficient experience may lead to specific complications, such as anastomotic leakage, gas leakage, or other pelvic injuries (56–58). This surgery requires advanced equipment, team collaboration, and surgical room resources, making it difficult to implement in many small to medium-sized medical institutions. With the establishment of standardized operating procedures and training systems, and the development of robotic-assisted technologies (59), more and more surgeons will be able to master this technique, making TaTME an indispensable part of rectal cancer surgery in the future.

## 5 Specimen extraction strategies

Natural Orifice Specimen Extraction (NOSE) is an innovative minimally invasive technique that allows surgical specimens to be extracted through natural orifices, such as the anus, eliminating the need for large incisions typically required in traditional surgeries. This approach reduces postoperative pain, wound complications, and the use of analgesics, while promoting faster recovery of intestinal function and shortening hospital stays (60, 61). The integration of NOSE with techniques such as TaE-ISR and RISR has introduced a revolutionary method for sphincter-preserving surgery in ultra-low rectal cancer. Recently, a “precision functional sphincter-preserving surgery” (PPS) approach has been proposed as an integrated workflow based on transanal NOSE. In patients with ultra-low rectal cancer, dedicated instruments are used to enable precise management of the distal tumor margin while preserving as much non-tumorous healthy rectal tissue as possible, and a hand-sewn anastomosis with mattress sutures is performed to enhance anastomotic reliability. Early findings suggest potential benefits in terms of perioperative safety, reduced need for a diverting stoma, and functional outcomes. This approach has been reported to improve operative efficiency, decrease the demand for prophylactic diversion, reduce procedure-related costs, and avoid an abdominal incision (62). Despite these potential advantages, NOSE as a specimen-extraction strategy still faces several challenges (63). The technique limits the visualization and operational space within the pelvic cavity, increasing the difficulty of surgical manipulation. These techniques involve steep learning curves and require surgeons to master advanced endoscopic or robotic skills to ensure accurate anatomical separation while avoiding damage to surrounding tissues, including nerves and blood vessels (64). Surgeons must be adequately prepared to address these inherent challenges, and continuous training and experience accumulation are essential for improving technical outcomes and functional recovery. As these techniques evolve, they hold immense potential to improve the quality of life for patients with low rectal cancer while achieving oncological success.

## 6 Multimodal strategies to enable sphincter/organ preservation: neoadjuvant therapy

In the management of ultra-low rectal cancer, the main neoadjuvant strategies include radiotherapy, chemotherapy, combined chemoradiotherapy, as well as targeted and immunotherapeutic approaches. The core value of neoadjuvant treatment is not merely “tumor shrinkage”; rather, by downstaging the tumor and controlling margin-related risks, it can increase the feasibility of sphincter-preserving procedures (e.g., uLAR/CAA and ISR) and, in a subset of patients who achieve substantial tumor regression, create opportunities for organ-preservation strategies. For locally advanced rectal cancer (LARC), neoadjuvant therapy has become an essential component of standard multimodal treatment, especially those patients with clinical staging of cT3 to cT4, no distant metastasis (M0), and regardless of lymph node positivity (cN+) (65), or when the tumor is located near the anal verge or is too large to be completely resected.

Neoadjuvant chemoradiotherapy strategies commonly include long-course chemoradiotherapy (CRT) and short-course radiotherapy (SCRT) combined with chemotherapy (65). Available studies indicate that neoadjuvant chemoradiotherapy can facilitate tumor regression and downstaging, including complete regression (TRG4, defined as complete disappearance of the tumor according to the Dworak system) as well as moderate regression (TRG 3 + 2). Such effects not only improve disease-free survival (DFS) and overall survival (OS), but also significantly increase resectability, enhance local control and long-term survival, and ultimately expand the likelihood of sphincter preservation (66–72). In ultra-low rectal cancer, tumor shrinkage and mitigation of distal tumor extension may reduce the technical difficulty of distal transection and reconstruction and, under strict control of circumferential resection margin (CRM) and distal margin safety, increase the feasibility of sphincter-preserving surgery (e.g., ISR) (73–75). Importantly, neoadjuvant treatment should be tightly integrated with surgical strategy; its purpose is to enable sphincter preservation more safely, rather than being discussed in isolation from procedural selection.

Total neoadjuvant therapy (TNT) has emerged as a treatment paradigm that has attracted considerable attention in recent years. By integrating chemoradiotherapy with additional systemic treatment, TNT delivers a comprehensive preoperative regimen and has been associated with higher rates of pathological complete response (pCR) compared with standard approaches (76–78). Treatment response varies substantially among patients, and a subset may fail to achieve meaningful regression (76). Accordingly, for patients with ultra-low rectal cancer, a response-adapted stratification may be applied: patients achieving a clinical complete response (cCR) or near–clinical complete response (near-cCR) may be considered for organ-preservation strategies within a framework of strict selection and intensive surveillance; patients with partial response but improved margin conditions and no involvement of the sphincter complex may be considered for sphincter-preserving resection and reconstruction (e.g., uLAR/CAA or ISR); whereas those with inadequate response, persistently threatened margins, or suspected invasion of the sphincter should prioritize oncologic safety and proceed with a more radical surgical strategy consistent with curative intent (78, 79).

In terms of precision therapy, immune checkpoint inhibitors—such as PD-1 inhibitors (e.g., pembrolizumab)—have demonstrated greater therapeutic sensitivity in patients with mismatch repair deficiency (dMMR) and high microsatellite instability (MSI-H), providing a new direction for exploration in the neoadjuvant setting. However, optimization strategies for the broader MSS/pMMR population still largely depend on combination regimens and ongoing clinical trials (80–83) Targeted agents against key oncogenic pathways, such as cetuximab and panitumumab, have been widely used in metastatic rectal cancer and have shown some efficacy in the neoadjuvant setting. Overall, neoadjuvant therapy should be implemented within a multidisciplinary framework, with careful weighing of treatment-related toxicities (e.g., myelosuppression and gastrointestinal adverse effects). Its ultimate goal is to provide patients with ultra-low rectal cancer opportunities for sphincter and organ function preservation—while ensuring oncologic safety—through improved local control and risk-adapted management (72, 76, 84–86).

## 7 Outlook for the future

In the future, with the further application of artificial intelligence, big data, and multimodal diagnostic technologies, treatment plans will become more refined. This will not only allow for accurate preoperative assessment of the patient’s condition but also enable dynamic monitoring postoperatively to detect risks of local recurrence or micro-metastasis in a timely manner, providing patients with safer, more effective, and personalized treatment options. Although gene therapy and gene editing techniques are still in the early stages of exploration for rectal cancer, their prospects are promising. This multi-layered, multi-perspective comprehensive treatment approach is gradually pushing rectal cancer treatment into a new stage.
